# Beyond linear Thinking: Redefining chemical pollution impacts on biodiversity

**DOI:** 10.1016/j.ese.2025.100589

**Published:** 2025-06-15

**Authors:** Yingying Liu, Xiaowei Jin, Aibin Zhan, Jinbao Liao, Andrew C. Johnson, Jian Xu

**Affiliations:** a State Key Laboratory of Environmental Criteria and Risk Assessment, Chinese Research Academy of Environmental Sciences, Beijing, 100012, China; b College of Water Sciences, Beijing Normal University, Beijing, 100875, China; c China National Environmental Monitoring Centre, Beijing, 100012, China; d Research Center for Eco-Environmental Sciences, Chinese Academy of Sciences, Beijing, 100085, China; e State Key Laboratory for Vegetation Structure, Function and Construction (VegLab), Ministry of Education Key Laboratory for Transboundary Ecosecurity of Southwest China, Institute of Biodiversity, School of Ecology and Environmental Science, Yunnan University, Kunming, 650091, China; f UK Centre for Ecology and Hydrology, Wallingford, Oxfordshire, OX10 8BB, UK

Since the onset of the Anthropocene, chemical pollution has emerged as a primary global threat to biodiversity across all biogeographical realms. This planetary-scale challenge affects ecosystem functionality from local to global scales, contributing significantly to biodiversity loss worldwide [1]. Traditional ecological risk assessments have predominantly relied on chemical-by-chemical dose–response linear models, presuming that incremental increases in harmful chemical pollutant concentrations lead to proportional declines in species abundance (Fig. 1a). However, this linear paradigm, which forms the foundation of environmental regulations across diverse geopolitical contexts—critically oversimplifies the intricate interactions within ecosystems. Such simplification fails to capture the multifaceted responses elicited by chemical pollutants interacting with other global change drivers across different biomes, ecoregions, and latitudinal gradients. Emerging evidence from cross-continental studies highlights that pollutant impacts on ecosystems often exhibit significant nonlinear characteristics, including thresholds, hysteresis, and potentially irreversible regime shifts [2]. These nonlinearities are shaped by ecological phenomena, including baseline stress levels, species sensitivity, and habitat connectivity. For example, pollutants that seem benign in isolation may cause severe disruption when associated with thermal stress or habitat loss [1]. Such nonlinear responses could be linked to delays in population recovery, spatial heterogeneity, adaptive traits, and reinforcing loops. For instance, in coral reef ecosystems, functional groups of herbivores, such as grazers and scrapers, play a critical role in mediating algal-coral dynamics and influencing ecosystem recovery trajectories [3]. These complex dynamics challenge traditional predictive frameworks and underscore the need for monitoring systems capable of detecting indirect, delayed, and context-dependent effects.

**Fig. 1:**
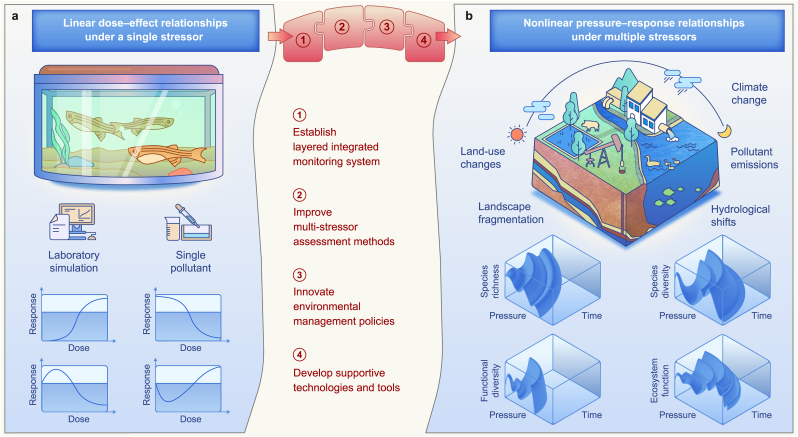
Frameworks of ecological risk assessments. **a**, Traditional dose–response linear models for conventional approaches. **b**, Nonlinear response patterns framework for natural ecosystems under multiple stressors.

Biodiversity across global ecosystems experiences nonlinear impacts from chemical pollutants. At sublethal levels prevalent in human-dominated landscapes, they subtly disrupt physiology, metabolism, and gene expression, thereby reducing individual fitness, reproduction, and population resilience, while also heightening susceptibility to co-stressors across diverse taxa. These effects accumulate through trophic levels and regions, gradually compromising ecosystem integrity without necessarily triggering detectable population declines. As concentrations rise, sensitive species may exhibit non-monotonic population dynamics, potentially triggering cascading disruptions via altered competition interactions, predation pressures, and ecosystem engineering processes [4]. Such threshold-driven responses, as emphasized by Folke et al. [3], reveal that minor increases in pollutant levels can precipitate disproportionate ecological disruptions, necessitating a review of context-specific safety thresholds to avert irreversible tipping points.

Chemical contaminants persist and bioaccumulate across interconnected ecosystems, posing significant threats to global biodiversity and ecosystem stability [5]. These pollutants interact with ecological drivers across spatial scales, including habitat structure, resource availability, disturbance regimes, and biotic interactions, shaping complex, multi-peaked biodiversity patterns that challenge linear and unimodal models across biomes [6]. This complexity demands a shift from simplistic risk assessments as we become aware of nonlinear interactions between pollutants and global change drivers across terrestrial, freshwater, and marine ecosystems. For instance, Rockström et al. [1] demonstrated that an agricultural nitrogen surplus of 61 Tg N per year, combined with land-use change and a tightened boundary of 57 Tg N per year due to groundwater nitrate, disrupted soil microbial communities and plant species richness in temperate grasslands. This case underscores how nutrient pollution and land degradation can interact through altered nutrient cycling and habitat loss, amplifying ecological impacts beyond those predicted by single-stressor models. Similarly, Schartup et al. [4] revealed non-additive interactions among climate warming, overfishing, and methylmercury (MeHg) bioaccumulation in the Gulf of Maine over three decades. A 1 °C rise in seawater temperature increased MeHg concentrations in Atlantic cod by 32 %, whereas overfishing-induced trophic shifts reduced them by 12 %, resulting in a net 10 % decrease. However, warming and herring depletion drove a 70 % surge in MeHg in spiny dogfish through physiological and dietary changes, highlighting the unpredictable nature of multiple interacting stressors over decades of environmental change in marine ecosystems. At a broader landscape scale, Johnson et al. [7] employed a machine learning model incorporating 41 environmental variables to explain 73 % of macroinvertebrate family richness variation in English rivers. Their findings revealed that elevated zinc and copper levels, particularly when combined with high wastewater exposure, disproportionately drove biodiversity declines, even after adjusting for habitat quality and hydromorphology, emphasizing the dominant influence of chemical stressors in freshwater ecosystems worldwide. These cases collectively demonstrate that the ecological risks of chemical pollution cannot be solely predicted by contaminant concentrations but must account for interactions with environmental context and co-occurring stressors. In stressed or degraded ecosystems, ranging from tropical to temperate zones, such synergies can amplify toxicological impacts, driving severe and potentially irreversible biodiversity and functional losses across multiple scales [3]. Addressing these global challenges, identifying and quantifying these nonlinear relationships, alongside defining ecological safety thresholds for pollutants, could be vital for advancing environmental science in the Anthropocene [1].

The intrinsic complexity and interconnectedness of ecological networks amplify the nonlinear effects of chemical pollutants, extending their impacts far beyond direct toxicological interactions across ecosystem boundaries. Pollutants influence ecosystems through multiple pathways, including bioaccumulation, biomagnification, food web restructuring, altered competitive dynamics, and delayed demographic responses, resulting in complex, nonlinear patterns at both community and ecosystem scales [3]. For example, biomagnification of persistent organic pollutants within food webs can exert selective pressure on apex predators through reproductive impairment or immune suppression. These disruptions cascade through trophic networks, potentially triggering alternative stable states characterized by fundamentally different community structures and ecosystem functions [9]. Additionally, ecosystems under multiple anthropogenic stressors become increasingly vulnerable to ecological tipping points, which are thresholds that trigger rapid, nonlinear shifts to alternate states with reduced biodiversity and ecosystem services [1,10]. These transitions, such as shallow lakes transitioning to phytoplankton-dominated systems or coral reefs collapsing into algal dominance, occur when interacting stressors disrupt feedback mechanisms [2,3]. Such disruptions arise from interactions between chemical pollution and other global environmental pressures, such as climate change and habitat fragmentation, which erode resilience by weakening negative feedback and amplifying positive ones, thereby accelerating shifts while hindering recovery. The combined effects of various environmental stressors, including chemical pollutants, climate change, and land-use change, heighten the probability of crossing these tipping points across ecosystems worldwide. For instance, the accumulation of persistent pollutants can undermine coral reef resilience, diminishing their capacity to withstand ocean acidification and accelerating their transition to degraded states [3]. These global cases underscore the urgent need for a shift from single-stressor assessments to integrated frameworks that capture complex, nonlinear dynamics in real-world ecosystems under multiple pressures.

Addressing these complex global challenges requires a fundamental paradigm shift toward a comprehensive framework centered on “nonlinear response patterns under multiple stressors” (Fig. 1b). We propose an innovative and integrated framework that consolidates multiple approaches to tackle the complex, multi-peaked nonlinear behaviors arising from interactions between chemical pollutants and biodiversity across ecosystems worldwide. Unlike traditional approaches, which often treat stressors in isolation, this framework considers the combined effects of multiple environmental stressors, providing a more holistic and predictive tool for ecosystem management in diverse geographical contexts [4,8]. It aims to overcome the limitations of conventional monitoring by integrating real-time data, advanced data analysis, predictive modeling, and technology development. To tackle chemical pollution's biodiversity impact across global ecosystems, this approach requires recognizing the complexity of anthropogenic chemicals and leveraging expertise from ecotoxicology, community ecology, biogeochemistry, and environmental informatics. Transdisciplinary collaboration strengthens predictive power and supports targeted, context-specific mitigation [1,2].

The framework comprises four interconnected components. First, a hierarchically structured, integrated monitoring system should be developed, combining chemical, biological, and ecological data to track pollutant effects across ecosystems [5]. Tools such as non-target screening (NTS), molecular biomarkers, and environmental deoxyribonucleic acid metabarcoding can detect emerging contaminants, shifts in community structure and function, and early warning signals of ecological transitions. In Chebei Stream, Guangzhou, NTS-based chemical fingerprints effectively traced pollutant sources in complex mixtures, illustrating its potential in ecosystem monitoring [8]. A multi-source data fusion platform can synthesize such data to identify tipping points and guide proactive management across environmental gradients. Second, multi-stressor assessments require advanced methods to quantify interactive effects across ecosystems. Tools such as mixture toxicity testing, food web metrics, and functional redundancy analysis help identify the impacts and thresholds of compounds. Machine learning enhances early warning capabilities by detecting signals such as critical slowing. At the global level, the Safe and Just Earth System Boundaries framework applies similar principles, revealing that multiple thresholds have been exceeded in densely populated regions [1]. Third, environmental management policies must integrate multi-stressor frameworks into chemical management decisions to boost adaptive capacity and ecological relevance. For instance, the European Union's Registration, Evaluation, Authorisation, and Restriction of Chemicals requires manufacturers to provide safety data before market approval, creating a strategic point for integrating nonlinear, multi-stressor monitoring [9]. Embedding real-time early warning systems based on remote sensing and biosensors into such frameworks can enable timely responses near ecological thresholds and improve resilience under multiple pressures. Finally, advancing supportive technologies is essential for nonlinear ecological risk assessment. Smart biosensors enable the real-time detection of stress in sentinel species, while remote sensing facilitates large-scale resilience monitoring. In the Amazon Basin, satellite vegetation indices showed increasing autocorrelation and slower recovery after droughts, indicating a decline in ecosystem resilience [10]. Combined with machine learning, these tools enhance the early detection of ecological tipping points and support timely interventions in response to multiple stressors. This framework lays a foundation for assessing the impacts of complex pollutants and setting science-based safety boundaries, thereby strengthening environmental decision-making amid interacting global change drivers.

The implementation of nonlinear ecological risk assessment at scale may face practical challenges, including high costs, data heterogeneity, and limited infrastructure, particularly in resource-constrained settings. These obstacles can be addressed through modular monitoring, standardized protocols, and cross-sector collaboration. While nonlinear models offer valuable insights, they are sensitive to data quality, structural assumptions, and system complexity, often producing outputs that are difficult to interpret or validate. Therefore, they are most effective when used as adaptive decision-support tools, supported by empirical validation and transparent communication of uncertainties. By integrating multidimensional monitoring with nonlinear analyses, environmental management can enhance the early detection of ecosystem instability and facilitate timely interventions. Ultimately, this framework fosters adaptive, context-specific risk models that identify ecological tipping points and pollution thresholds, thereby enhancing policy responses, safeguarding biodiversity, and sustaining ecosystem resilience in the face of global change.

## CRediT authorship contribution statement

**Yingying Liu:** Writing – original draft, Investigation. **Xiaowei Jin:** Writing – review & editing, Methodology, Conceptualization. **Aibin Zhan:** Methodology, Investigation. **Jinbao Liao:** Methodology. **Andrew C. Johnson:** Validation, Methodology. **Jian Xu:** Writing – review & editing, Conceptualization.

## CRediT authorship contribution statement

**Yingying Liu:** Writing – original draft, Investigation. **Xiaowei Jin:** Writing – review & editing, Methodology, Conceptualization. **Aibin Zhan:** Methodology, Investigation. **Jinbao Liao:** Methodology. **Andrew C. Johnson:** Validation, Methodology. **Jian Xu:** Writing – review & editing, Conceptualization.

## Declaration of competing interest

The authors declare that they have no known competing financial interests or personal relationships that could have appeared to influence the work reported in this paper.
